# Rheumatism

**Published:** 1906-04-28

**Authors:** 


### Progress in Medicine and Surgery.

### RHEUMATISM.

**Rheumatic Chorea.**—Theories as to the origin of chorea have followed one another in an endless succession, and the recent proofs of its rheumatic origin were contradicted almost as soon as they were published. It is of course difficult to imagine two diseases more unlike than chorea and acute articular rheumatism, and the frequent occurrence of rheumatism either before or after chorea in the same patient is not a matter on which accurate statistics are easily obtainable, nor if they were, would it prove that the two diseases are due to the same cause. However, other arguments in favour of a rheumatic origin through the diplococcus rheumaticus are not easily overturned. The proofs that the diplococcus is the cause of acute rheumatism go far to satisfy Koch's canons. Thus Poynton and Payne have isolated it in 32 cases, and many other observers have done so too. Secondly, they have cultivated it in pure growths, and they have reproduced most of the symptoms of the disease in rabbits and monkeys by injection of these cultures; and lastly, no other organism has been found regularly or even commonly in rheumatism. What then has led to the denial of its claims and the failure of some authors to find it? As a matter of fact it is not easy to discover in the blood, though occasionally this has been done, nor in the joint exudate, though this is possible when the type of the disease is malignant or the lining of the joint is much damaged. The organism indeed seeks the areolar tissue below the free margin of the synovial cavity, and is quickly destroyed by leucocytes if it escapes into the fluid. If looked for in the right place there seems no difficulty in finding it. But others say, Is the organism you find always one and the same? The authors confess it is difficult to find a specific test for it, as indeed for the typhoid bacillus, and so to show that it does not occur in any other disease; but they point out that it is smaller than the streptococcus pyogenes, and that it survives drying even for as much as six months. It evolves more acid than this streptococcus, and at an earlier period. One of the acids at least which it produces is formic acid, and it will grow in a filtered culture of the streptococcus, a clear proof of its diversity. Moreover, rabbits are more resistant to it, and it never produces secondary abscesses in them or staining of the blood-vessels as the streptococcus does. No other organism is so nearly akin to it as the streptococcus, and we see that even that has strongly marked points of difference. Thus there is good ground for the claim that it is a specific organism and the cause of acute rheumatism, and we may add of malignant endocarditis. What then is its relationship to chorea? Now it is quite possible that some cases are due to other causes, but the great majority are probably manifestations of rheumatism and the symptoms due to a form of meningitis; since this diplococcus has been isolated in the cerebrospinal fluid in fatal cases of rheumatism in which chorea was present, and Payne and Poynton have produced twitching movements in rabbits by injecting the diplococcus, and again isolated it from the brain and pia mater of those rabbits, and they have recently found it in the same locality in a case of chorea when symptoms of acute rheumatism were absent. It is probable then that rheumatic hyperpyrexia is due to a general toxæmia, chorea to slight cerebral lesions, and between the two we may place some cases of rheumatic meningitis. How the diplococcus acts on the brain in producing the spasmodic movements of chorea is another question; whether it causes occlusion of the vessels, or whether its formic acid and other products directly irritate the brain substance is open to discussion; but there is some evidence that it does under some conditions cause a direct inflammation of the walls of the vessels. Thus Barie has described arteritis as occurring in acute rheumatism. It usually affects the aorta, but may attack the peripheral vessels or the coronary arteries. There is pain localised in the course of the vessel with heaviness and weakness in the limb, which may be cold and swollen. In a few instances there is pyrexia, and the artery becomes obliterated, though no case of gangrene has been reported. In mild attacks the symptoms clear up in the second week. Occasionally arterio-sclerosis follows, and where coronary vessels are affected heart failure or fatal results may ensue. It is perhaps remarkable that arteritis is so rare and endocarditis so frequent. If the former does occur in the brain it may be the means through which choreic movements are excited. Important questions as to treatment arise if we acknowledge that chorea is due in some way to the diplococcus. As the organisms are rapidly destroyed in the blood or joint exudates by the leucocytes, the salicylic compounds may owe some of their efficiency to the leucocytosis which they cause, but they probably also neutralise the toxins. However, the action of the salicylates on the pyrexia and joint symptoms is fleeting unless they are given for a considerable period, so that it is doubtful whether they have any direct bactericidal action on the micrococci deeply imbedded in the tissues. Again, they have comparatively little curative effect on endocarditis or chorea. Alkaline treatment of rheumatism does have a preventative action to some extent over endocarditis, lessening the frequency of its occurrence, but evidence is wanting as its value in preventing chorea. It would be interesting to compare the frequency of chorea in rheumatic cases treated by salicylates with those treated by alkalies.

*(To be continued.)*

